# Effect of niche components on masseter satellite cell differentiation on fibrin coatings

**DOI:** 10.1111/eos.12849

**Published:** 2022-01-12

**Authors:** Olivier Willem Lijten, Doris Haydee Rosero Salazar, Merijn van Erp, Ewald Bronkhorst, Johannes W. Von den Hoff

**Affiliations:** ^1^ Department of Orthodontics and Craniofacial Biology Radboud Institute for Molecular Life Sciences Radboud University Medical Center Nijmegen The Netherlands; ^2^ Department of Biochemistry Radboud Institute for Molecular Life Sciences Radboud University Medical Center Nijmegen The Netherlands; ^3^ Department of Dentistry Radboud University Medical Center Radboud Institute for Health Sciences Nijmegen The Netherlands; ^4^ Department of Medical Basic Sciences Faculty of Health Universidad Icesi Cali Colombia

**Keywords:** cleft palate, entactin, hydrogel, in vitro, laminin, skeletal muscle

## Abstract

In skeletal muscles, niche factors stimulate satellite cells to activate and induce muscle regeneration after injury. In vitro, matrigel is widely used for myoblast differentiation, however, is unsuitable for clinical applications. Therefore, this study aimed to analyze attachment and differentiation of satellite cells into myotubes on fibrin coatings with selected niche components. The attachment of satellite cells to fibrin alone and fibrin with niche components (laminin, collagen‐IV, laminin‐entactin complex [LEC]) were compared to matrigel. Only on matrigel and fibrin with LEC, Pax7‐positive cells attached well. Then, LEC was selected to analyze proliferation, differentiation, and fusion indices. The proliferation index at day 1 on fibrin‐LEC (22.5%, SD 9.1%) was similar to that on matrigel (30.8% [SD 11.1%]). The differentiation index on fibrin‐LEC (28.7% [SD 6.1%] at day 5 and 32.8% [SD 6.7%] at day 7) was similar to that on matrigel (40.1% [5.1%] at day 5 and 27.1% [SD 4.3%] at day 7). On fibrin‐LEC, the fusion index at day 9 (26.9% [SD 11.5%]) was similar to that on matrigel (25.5% [SD 4.7%]). Our results showed that the addition of LEC enhances the formation of myotubes on fibrin. Fibrin with LEC might be suitable to enhance muscle regeneration after surgery such as cleft palate repair and other muscle defects.

## INTRODUCTION

Cleft lip and/or palate (CL/P) is the most common craniofacial birth defect with a worldwide prevalence of about 1.7 in 1000 newborns [1]. The CL/P malformations vary from a unilateral or bilateral cleft of only the upper lip to a complete cleft of the palate, alveolus, and soft palate [1]. The oral and the nasal cavities are connected, which causes problems for eating and speaking [2]. After surgical treatment, soft palate dysfunction and speech problems persists in 20–30% of the cases, of which the main cause is muscle fibrosis and impaired muscle regeneration [3, 4]. Fibrosis and impaired muscle regeneration also occur in other extensive muscle injuries such as in severe trauma and disease [5].

In general, skeletal muscles have the ability to regenerate through the action of their stem cells also known as satellite cells [6, 7]. Satellite cells are located around the myofibers and are involved in postnatal muscle growth and muscle repair after injury [8]. Muscle development and muscle regeneration share similar processes and transcription factors [9]. Upon injury, satellite cells proliferate, differentiate and fuse to form new myofibers and repair the injured ones [6]. Muscle healing occurs through three phases: inflammation, regeneration, and remodeling [10]. Activation and proliferation of satellite cells occur in the regeneration phase when paired‐box protein‐7 (Pax‐7)‐positive satellite cells start to express myogenic regulatory factors (MRF) [9, 10, 11]. The initial step of myogenesis is followed by the expression of myogenic differentiation factor (MyoD), myogenin (MyoG), and MRF4 [9, 10, 11]. The myoblasts then fuse to form myotubes that turn into mature myofibers expressing myosin heavy chain (MyHC) and other contractile proteins [9, 10, 11]. The muscles of the head and neck, or branchiomeric muscles, contain lower numbers of satellite cells and develop more fibrosis after injury, which reduces their capacity to regenerate [12, 13].

The activity of satellite cells mainly depends on their microenvironment, the niche. The basement membrane is the structural part of the niche, containing laminin and collagen type IV linked by entactin, that provides attachment sites, but also serves as a reservoir of growth factors [14]. The basement membrane together with the adjacent myofiber form the niche of the satellite cells where chemical, mechanical, and electrical signals regulate their activity [6]. Strategies to improve muscle regeneration include the use of stem cells, growth factors, and biomaterials for implantation. Satellite cells, mesenchymal stem cells, and embryonic stem cells are the most commonly implanted types of cells in regenerative medicine for muscle tissue [15]. Challenges for the implantation of stem cells include the long‐term survival of the cells and adverse immune reactions [16, 17, 18]. Therefore, it might be more suitable to apply biomaterials without cells that contain basement membrane components to guide muscle regeneration by local satellite cells [13]. An often‐used biomaterial for tissue engineering is fibrin [15, 19‐29]. Fibrin is a biopolymer of the monomer fibrinogen. Fibrin forms after thrombin‐mediated cleavage of fibrinopeptide A and fibrinopeptide B, the two peptide chains of the fibrinogen‐molecule [30]. Upon wounding, fibrin forms a clot that seals the wound to prevent further bleeding [23, 31].

Fibrin is a biomaterial used in vitro and in vivo for research into heart and skeletal muscle regeneration [26, 32, 33]. Also, fibrin sealant is widely used in multiple surgical procedures [34, 35]. Thus, fibrin seems to be a promising material in regenerative medicine suitable for clinical use. However, satellite cells delivered in fibrin constructs often fail to differentiate unless basement membrane components are added [36, 37]. Basement membrane components such as laminin‐111 mixed with fibrin stimulate myofiber formation in vivo and so in vitro laminin‐entactin mixed with collagen [38, 39]. Then, in the present study, fibrin was evaluated as a coating for myoblast differentiation and myotube formation. Fibrin was compared to matrigel, a hydrogel that contains the basement membrane components laminin, collagen IV, and entactin [14, 40, 41, 42, 43]. Despite the favorable characteristics of matrigel, it is unsuitable for clinical use because it is derived from the Engelbret‐Holm‐Swarm (EHS) mouse tumor cell line that may present a risk of tumor development. Therefore, the aim of this study is to analyze the effects of fibrin with and without muscle basement membrane components on myotube formation in vitro. This knowledge will be useful for the development of fibrin‐based strategies for the improvement of muscle regeneration in the soft palate of CL/P patients and other skeletal muscle defects.

## MATERIAL AND METHODS

### Animals

Male wistar rats, 9 weeks old, were housed two per cage and received food and water ad libitum under standard housing conditions with a light/dark cycle of 12 h. The experiments were approved by the Animal Experimentation Committee of the Radboud University (RU‐DEC‐2014‐187‐003) and the Central Animal Testing Committee (CCD) in accordance with Dutch laws and regulations.

### Satellite cell isolation and culture

Four animals were sacrificed by the standard CO_2_/O_2_ protocol. Next, the masseter muscles from both sides of the head were dissected and stored in phosphate‐buffered saline (PBS; Gibco) supplemented with penicillin/streptomycin 2% (Gibco). Satellite cells (SCs) were isolated as described in a previous publication [44]. In short, the muscles were minced and digested in Pronase 0.1% (Calbiochem, Sigma‐Aldrich) for 1 h at 37°C, pipetted up and down multiple times and centrifuged. The isolated cells were pooled, expanded for 3 d in culture medium and frozen in 1 mL vials for later use. Each masseter yields approximately 1 × 10^6^ cells [44]. After a 1‐day culture on matrigel, this cell population contained 80.0% [SD 13.8%] Pax7‐positive cells. SCs (2 × 10^3^) in 10 μL of culture medium were seeded onto the coatings in 96‐well plates (see below). The plates were placed in the incubator for 2 h at 37°C to allow attachment. After incubation, 200 μL of culture‐medium was added to each well. The culture medium was Dulbecco's modified Eagle's medium with 4,500 mg/L glucose, 4 mL L‐glutamine, and 110 mg/mL sodium pyruvate supplemented with 20% fetal bovine serum (v/v), 10% horse serum (v/v), 1% penicillin‐streptomycin, and 1% chicken extract embryo filtered through a 0.2 μm filter. This culture medium promotes both the proliferation as well as differentiation of myoblasts [44, 45]. The medium was changed every day. To analyze attachment to the coatings, SCs were cultured in triplicate for one day, then fixed and stained for analysis (see below). A selection of coatings was used to analyze cell proliferation, differentiation, and myotube formation in 9‐day cultures. Triplicate cultures were fixed and stained after 1, 5, 7, and 9 days. Matrigel coating was always used as a positive control.

### Coatings

To prepare the fibrin coating, fibrinogen (Sigma‐Aldrich) was reconstituted in 0.9% NaCl to a concentration of 40 mg/mL at 37⁰C and filtered through a 0.2 μm filter. Two hundred microliters Aprotinin (Sigma‐Aldrich) 1 mg/mL was diluted in 300 μL PBS and mixed with 500 μL fibrinogen solution (1:1). Thrombin (T7513 Sigma‐Aldrich, 10 U/mL), was reconstituted in 0.1% bovine serum albumin (BSA) and mixed with CaCl_2_ 10 mM (1:1). The coating solution had final concentrations of 5 mg/mL fibrinogen, 125 μl/mL aprotinin, 2.5 U/mL thrombin, and 2.5 mM CaCL_2_. Each well of a 96‐wells plate was coated with 10 μL of coating solution.

To prepare the matrigel coating (Corning Matrigel Basement membrane matrix, product no. 356237, phenol red‐free at standard growth factor concentration; Sigma‐Aldrich), 200 μL of the initial protein solution (8.6 mg/mL) was diluted in 1800 μL of DMEM‐HG (Dulbecco's modified Eagle's medium high glucose) containing 4500 mg/L glucose, 4 mL L‐glutamine, and 110 mg/mL sodium pyruvate (Gibco). The prepared matrigel was kept on ice for 15 min, and 10 μL was applied in each well with a pre‐chilled micro‐pipette. Afterward the plates were dried for 1 h at 37°C (95% air, 5% CO_2_).

Laminin, collagen IV, and laminin‐entactin were used as niche factors. These three components are part of the basal lamina of the myofibers and also present in matrigel [46, 47]. Laminin (LN521‐03 BioLamina) that binds to the integrins expressed by primary cells such as integrin α7β1 [48]. This component was used in concentrations of 16.7, 33.3, and 66.6 μg/mL in PBS. Collagen IV (C5533‐5MG, Sigma‐Aldrich) was also used in concentrations of 16.7, 33.3, and 66.6 μg/mL in PBS. For laminin‐entactin (high concentration, 354259‐Corning, Sigma‐Aldrich), the concentrations were 50, 100, and 200 μg/mL in PBS. The types of laminins in the complex are the LN111 and LN211, the same as in matrigel.

For fibrin plus laminin, the final concentrations were also 16.76 μg/mL (1), 33.3 μg/mL (2), and 66.6 μg/mL (3) in the fibrin mixture. The final concentrations for collagen IV plus fibrin were prepared in the same way. For laminin‐entactin complex with fibrin, the final concentrations were 50 μg/mL (1), 100 μg/mL (2), and 200 μg/mL (3) in the fibrin mixture.

### Immunofluorescence staining

For staining, the cells were washed with prewarmed PBS and fixed with 200 μL of neutral buffered formalin 10% for 10 min. The wells were washed in PBS again followed by incubation in 100 mM glycine in tris‐buffer (TBS, pH 7.4) for 30 min. The cells were permeabilized in 0.5% Triton X‐100 in TBS for 30 min and then washed in 0.05% Tween‐20 in TBS. Blocking buffer containing 5% normal goat serum, Triton X‐100 0.5%, Tween‐20 0.05% in TBS, was added to each well and incubated overnight at 4°C. Next, the first antibodies in blocking buffer were applied to each well; mouse anti‐human Pax7 (1:100, Developmental Studies Hybridoma Bank, Bioconnect), mouse anti‐human MyoG (1:100, F5D, Developmental Studies Hybridoma Bank, Bioconnect), or rabbit anti‐human MyoD (1:250 Sanbio) in blocking buffer. A double staining with antibodies against Pax7/MyoD or MyoG/MyoD was performed by mixing both antibodies. For MyHC staining, rabbit‐anti‐mouse fast‐MyHC (1:500, Sigma‐Aldrich) in blocking buffer was applied. After washing in 0.05% Tween‐20 in TBS, the secondary antibodies were applied. These were Alexa Fluor 488 goat‐anti‐mouse IgG (H+L, 1:1000, Invitrogen), Alexa Fluor 647 goat‐anti‐rabbit IgG (H+L, 1:1000, Invitrogen/Thermo‐Fisher), or Alexa Fluor 647 goat‐anti‐mouse IgG (H+L, 1:1000, Invitrogen) in blocking buffer. For double staining, a mixture of two secondary antibodies was added. For nuclear visualization DAPI (4′,6‐diamidino‐2‐phenylindole, 0.4 μg/mL) in PBS was applied for 10 min followed by rinsing in PBS and water. Lastly, 20 μL of mounting medium, glycerol 90%, 0.5% DABCO (1.4 Diazobicyclo‐(2,2,2)octane) in TBS, pH 8.6, was added to each well.

### Imaging, image analysis, and quantification

Multi‐channel 8‐bit images of the immunofluorescence staining were taken with a high content microscope (Leica DMI 6000B, Leica) at 10x magnification. The same settings of brightness, light exposure, and saturation were used for all images. Each well was photographed at five standardized spots; the center of the well and then above, beneath, at the left and at the right of the center. The image analysis was performed with Fiji (LOCI, University of Wisconsin) [49]. The automated quantification involved pixel recognition for nuclei counting. The average diameter of nuclei in muscle cells is 10 μm approximately [50]. The minimum size for nuclei detection was set at 15 pixels in the DAPI staining. Next, a macro sequence for Fiji was built for cell‐counting, staining‐detection and myotube recognition purposes. In order to objectively measure the number of nuclei we used the Fiji image processing package. A Fiji macro was created and automatically applied to all images.

The following processing pipeline was used on every image:

1. **Split channels**: Each antibody needs to be processed independently, so the multi‐channel images are divided into the separate channel images.
2. **Threshold**: For all the channel images using IsoData.
3. **Segment the nuclei**: In order to identify all nuclei, a watershed segmentation is performed on the DAPI channel image and all nucleus segments are counted. When counting, an acceptable nucleus size range of 15–230 pixels was used. Any segments outside the size range are considered artifacts and not used in further processing. The same range is applied to all collected images. Finally, any segment at the edge of the image is also disregarded to avoid any partial nuclei.
4. **Determining nucleus staining**: For every nucleus segment, the outline is projected on the green and red channel images. Then, for both channels, the macro checks if there are any segments within the nucleus outline. If so, the nucleus is counted as stained with the red or green marker, and double‐stained if both type of segments occur within the same nucleus. To avoid segment artifacts, the staining segments are restricted in size as well with the range being 15–230 pixels.

The proliferation index was calculated by dividing the number of Pax7‐MyoD double‐positive cells by the total number of Pax7‐positive cells and expressed as a percentage [51]. The differentiation index was calculated by dividing the number of MyoD‐MyoG double positive cells by the total number of MyoD‐positive cells and expressed as a percentage [51]. MyHC positive myotubes were also counted. The fusion index was calculated as the number of nuclei inside the myotubes divided by the total number of cells [51].

### Statistics

The Fiji macro was validated by manual counting of 16 random pictures in the blue, red, and green channels as well as the overlay of these. Statistics comparing matched observations were all based on paired t‐tests and Pearson correlations. Observer 1 (OL) scored a mean correlation between manual and automated counting of 0.901 (range 0.745‐0.997). Observer 2 (DR) scored a mean correlation between manual and automated counting of 0.900 (range 0.753‐0.996). Only for the overlay of channels, was a systematic difference between macro and manual counting found. Manual counting was on average 1.7 higher for Observer 1 (*p* = 0.047) and 2.2 higher for Observer 2 (*p* = 0.034). Given the small size of this differences as compared to a mean of approximately 40 for this outcome, and the excellent reproducibility for the macro count, with a duplicate measurement error of 0.18 for both observers, the macro counts were considered a reliable method.

The data from the 1‐day cultures were only used to select the best niche component for addition to fibrin. For the 9‐day cultures, we used a multivariable linear regression (R version 3.6.3) and analyzed the effect of type of coating, the effect of time and interactions simultaneously. Interactions between time and coating were only added when it resulted in a statistically significant improvement of the model. For all outcomes, the comparison between matrigel and the other coatings are presented as estimated on day 1, 5, 7, and 9 by the regression model. Only for myotube number and differentiation index the day 1 data were left out of the analysis as the low response at that point in time made a comparison superfluous. The graphs were all made in GraphPad Prism version 5 (GraphPad Software; www.graphpad.com). For all experiments, unless otherwise noted, *p* values less than 0.05 were considered significant (see Supporting Information for detailed data from the statistical analysis).

## RESULTS

### Attachment of satellite cells

**FIGURE 1:**
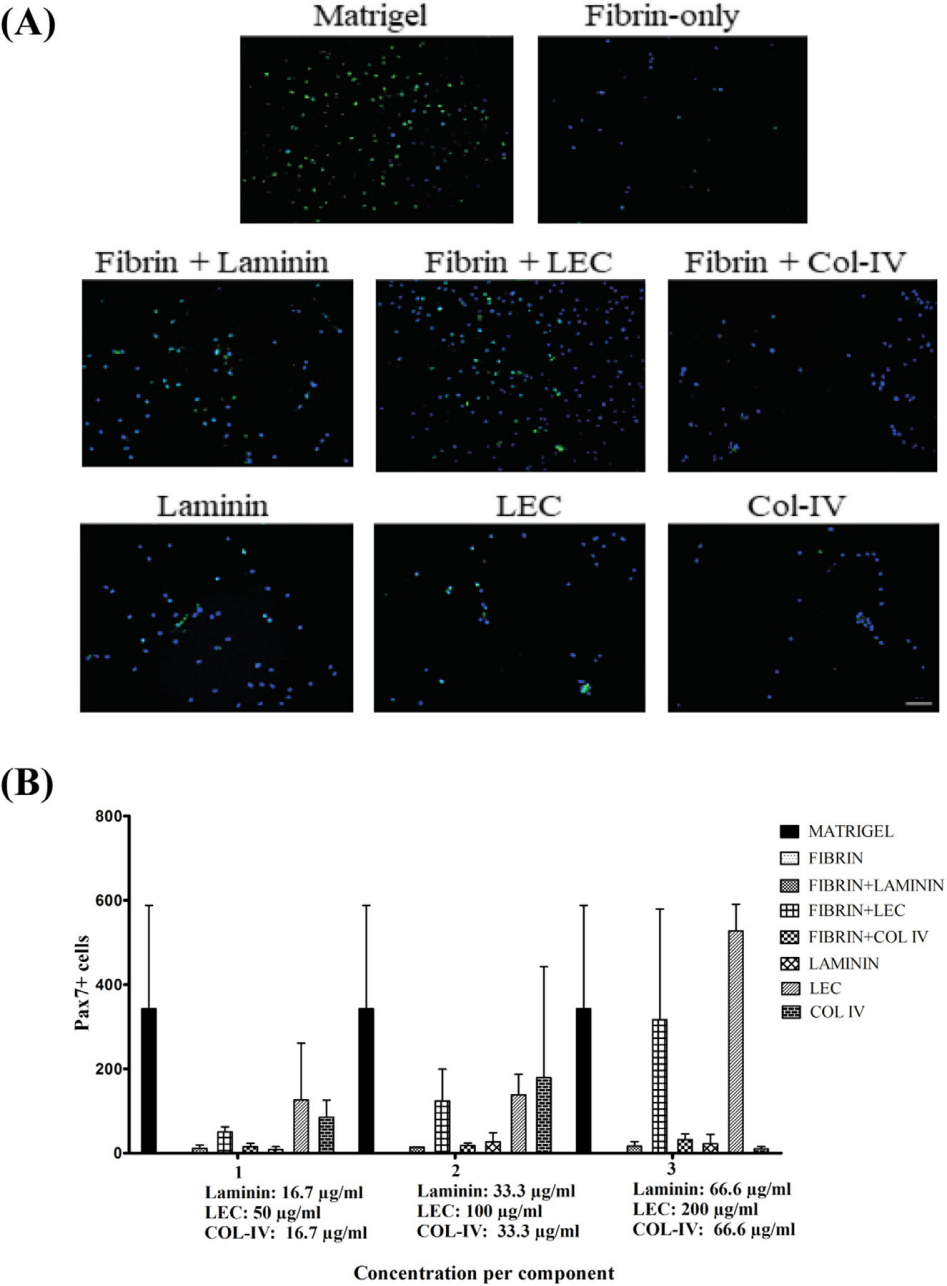
Attachment of satellite cells. (A) Representative images of murine satellite cells (SCs) derived from the masseter seeded either on matrigel, collagen IV (Col IV 33,3 μg/mL), laminin (33,3 μg/mL), laminin‐entactin complex (LEC, 100 μg/mL), fibrin alone, fibrin with laminin (33,3 μg/mL), fibrin with collagen IV (Col IV 33,3 μg/mL), and fibrin with laminin‐entactin complex (LEC, 100 μg/mL). Cells were fixated 24 h after seeding and stained with mouse anti‐human Pax7, Alexa fluor 488, and DAPI (blue). Scale bar represents 100 μm. (B) Numbers of Pax7 positive‐cells on the coatings. Laminin and collagen IV were used in concentrations of 16,7 μg/mL (1), 33,3 μg/mL (2), and 66,6 μg/mL (3). For laminin‐entactin complex, the concentrations were 50 μg/mL (1), 100 μg/mL (2), and 200 μg/mL (3). N = 3. Data are shown as mean and ± SD

Satellite cells (SCs) isolated from the masseter muscle were cultured for 24 h on matrigel, fibrin alone, fibrin with laminin, fibrin with collagen IV (Col IV), and fibrin with laminin‐entactin complex (LEC) in three different concentrations. Also, SCs were cultured on the niche‐factors alone. For comparison, matrigel coatings were taken as positive controls (Figure 1A). The images showed that on matrigel, fibrin with laminin and fibrin with LEC, many Pax7‐positive cells attached for later quantification. In contrast, a smaller number of positive cells attached to the other coatings (Figure 1A).

The mean number of Pax 7‐positive cells on matrigel was 342.3 (SD 245.6) while on fibrin alone a few cells attached that were not Pax7+. This may indicate that SCs attach less to fibrin than other cells such as fibroblast. The addition of laminin to fibrin did not increase cell attachment, nor did the addition of collagen IV in any of the concentrations. Fibrin plus LEC increased attachment to the level seen on matrigel in the two highest concentrations (124.0 [SD 75.0] in concentration 2, and 316.7 [SD 262.4] in concentration 3) that were not significantly different. Finally, the coatings with collagen IV alone and laminin alone showed lower cell attachment of Pax7+ cells than LEC alone coatings. Based on these results we chose fibrin plus LEC (concentration 2) for further experiments. Fibrin alone, LEC alone and matrigel were used for comparison (Figure 1B).

### Satellite cell differentiation

Satellite cells were cultured on matrigel, fibrin alone, fibrin plus laminin‐entactin, and laminin‐entactin alone. Cultures at days 1, 5, 7, and 9 were fixed and double‐stained for Pax7/MyoD to determine the proliferation index, and for MyoD/MyoG to determine the differentiation index. To determine myotube formation, the cells were stained for MyHC.

#### Pax7/MyoD double staining

**FIGURE 2:**
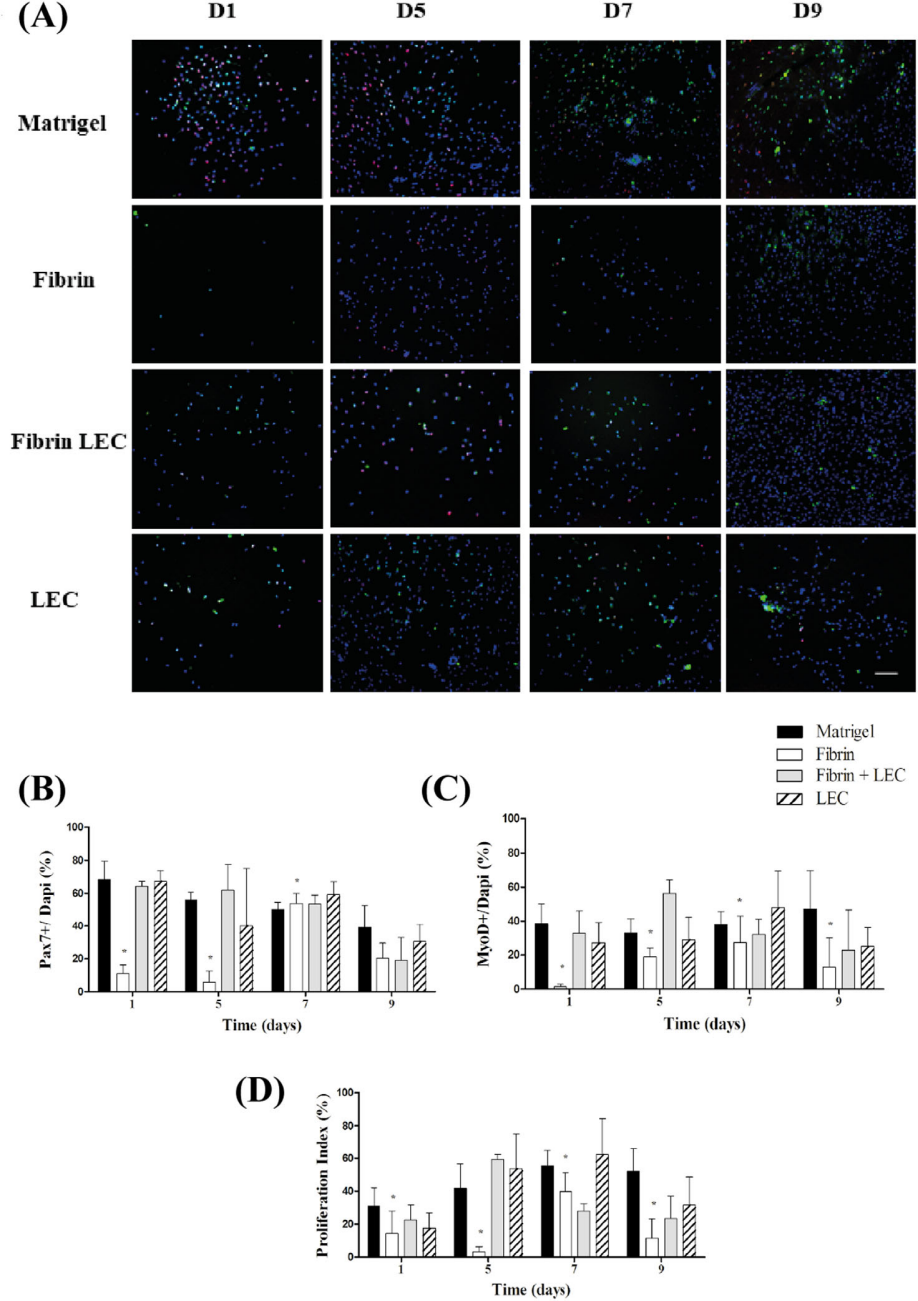
Pax7/MyoD double staining. (A) Representative Pax7 (green) and MyoD (red) double‐staining. Satellite cells (SCs) derived from the masseter were seeded on matrigel, fibrin, fibrin plus laminin‐entactin complex (LEC), and LEC alone. Cells were fixed on days 1, 5, 7, and 9 and double‐stained for Pax7 and MyoD. Blue is the nuclear staining (DAPI). The scale bar represents 100 μm. (B) Percentages of Pax7‐positive cells on the coatings, (C) percentages of MyoD‐positive cells on the coatings, and (D) proliferation index, calculated by dividing the number of double‐positive cells by the number of Pax7‐positive cells. * indicates a statistically significant difference for the coating compared to matrigel, according to the multiple regression analysis

Figure 2A shows an overview of the staining for Pax7 (green) and MyoD (red) for each timepoint per coating. At day 1, a high number of cells (DAPI) were present on the matrigel coating, while the fibrin coating showed a low number of cells. The number of cells on both fibrin + LEC and LEC alone seems to be initially lower than on matrigel. The total number of cells seems to increase on all coatings during culture.

The percentage of Pax7+ cells was quantified in all cultures (Figure 2B). Regression analysis resulted in a model with interaction between time and coating for Pax7+ (Table S1). For matrigel, the percentage of Pax7‐positive cells significantly decreased over time from 68.3% [SD 11.1%] to 39.1% [SD 13.5%] (*p* < 0.05). The percentage of Pax7‐positive cells on fibrin was significantly lower than on matrigel at days 1 (10.9% [SD 5.2%]), 5 (5.6% [SD 6.9%]) and 7 (53.9 % [SD 6.0%]) according to the model (all *p* < 0.05). The other coatings did not show differences with matrigel at any time point.

The percentage of MyoD‐positive cells was quantified in all cultures (Figure 2C). On matrigel, the percentage of MyoD‐positive cells was around 40% over time. There were no significant interactions with time. For fibrin alone, the percentage of MyoD‐positive cells was lower at all time points (*p* < 0.05) (Table S1). For fibrin plus LEC and LEC alone, the number of MyoD‐positive cells was similar to matrigel in time.

Finally, to estimate the rate of proliferation, a proliferation index was calculated by determining the percentage of Pax7‐positive cells that were also MyoD‐positive (Figure 2D). The proliferation index on matrigel increased from 30.8% [SD 11.1%] at day 1 to 52.0 % [SD 13.9%] at day 9. There were no significant interactions with time (Table S1). The proliferation index on fibrin at all timepoints was lower than on matrigel (*p* < 0.05). The proliferation index on the two other coatings was similar to matrigel.

#### MyoD/MyoG double staining

**FIGURE 3:**
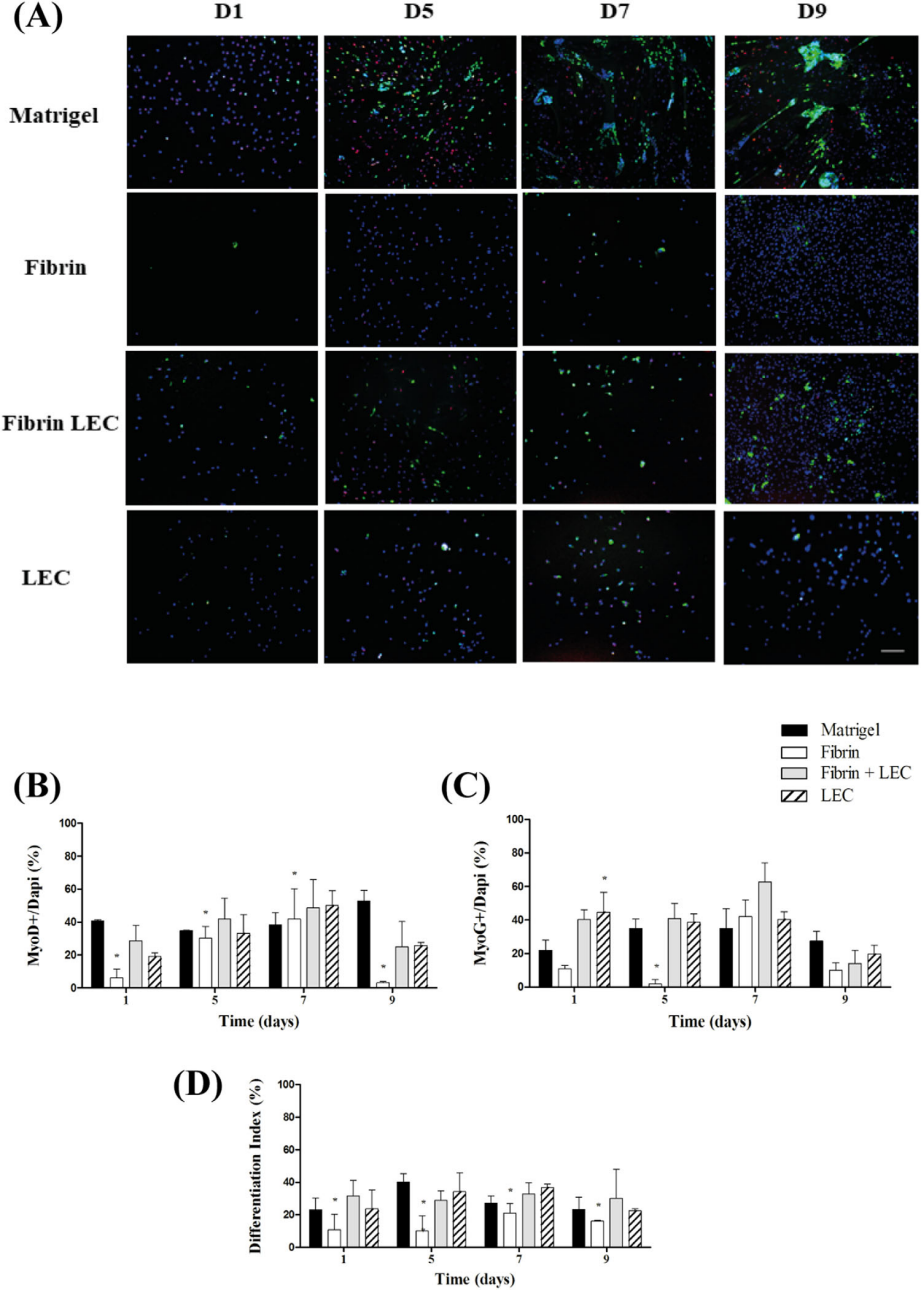
MyoD/MyoG double‐staining. (A) Representative MyoD (red) and MyoG (green) double‐staining. Murine satellite cells (SCs) derived from the masseter muscle were seeded on matrigel, fibrin, fibrin plus laminin‐entactin complex, and laminin‐entactin complex (LEC), and cultured up to 9 days. Blue is the nuclear staining (DAPI). Scalebar represents 100 μm. (B) Percentage of MyoD‐positive cells, (C) percentage of MyoG‐positive cells, and (D) differentiation index. * indicates a statistically significant difference for the coating as compared to matrigel, according to the multiple regression analysis

Initially, a low number of cells were present on the fibrin coatings compared with the other coatings (Figure 3A). The total number of cells (DAPI) for each coating seems to increase in time. For all coatings, the percentage MyoD‐ and MyoG‐positive cells seems to increase toward day 9.

The percentage of MyoD‐positive cells was quantified in all cultures (Figure 3B). The percentage of MyoD‐positive cells on matrigel appears to remain stable around 40%. There was no interaction with time (Table S2). On fibrin alone, the percentage MyoD‐positive cells was lower at all timepoints (*p* < 0.05). The other two coatings were both similar to matrigel.

The percentage of MyoG‐positive cells on matrigel was about 30% (Figure 3C). There was a significant interaction with time (Table S2). At day 1, the MyoG‐positive cells on LEC was higher than matrigel (*p* < 0.05). On fibrin alone at day 5, the number of positive cells was lower than matrigel (*p* < 0.05). At the other time points, all coatings were similar to matrigel.

At last, to estimate the differentiation rate, a differentiation index was determined by calculating the percentage of MyoD‐positive cells that were also MyoG‐positive (Figure 3D). There was no significant interaction with time (Table S2). The differentiation index on matrigel was between 20% and 40%. On fibrin alone, the differentiation index was lower at all timepoints (*p* < 0.05). For fibrin plus LEC and LEC alone, the differentiation index was similar to matrigel.

#### Myotube formation

**FIGURE 4:**
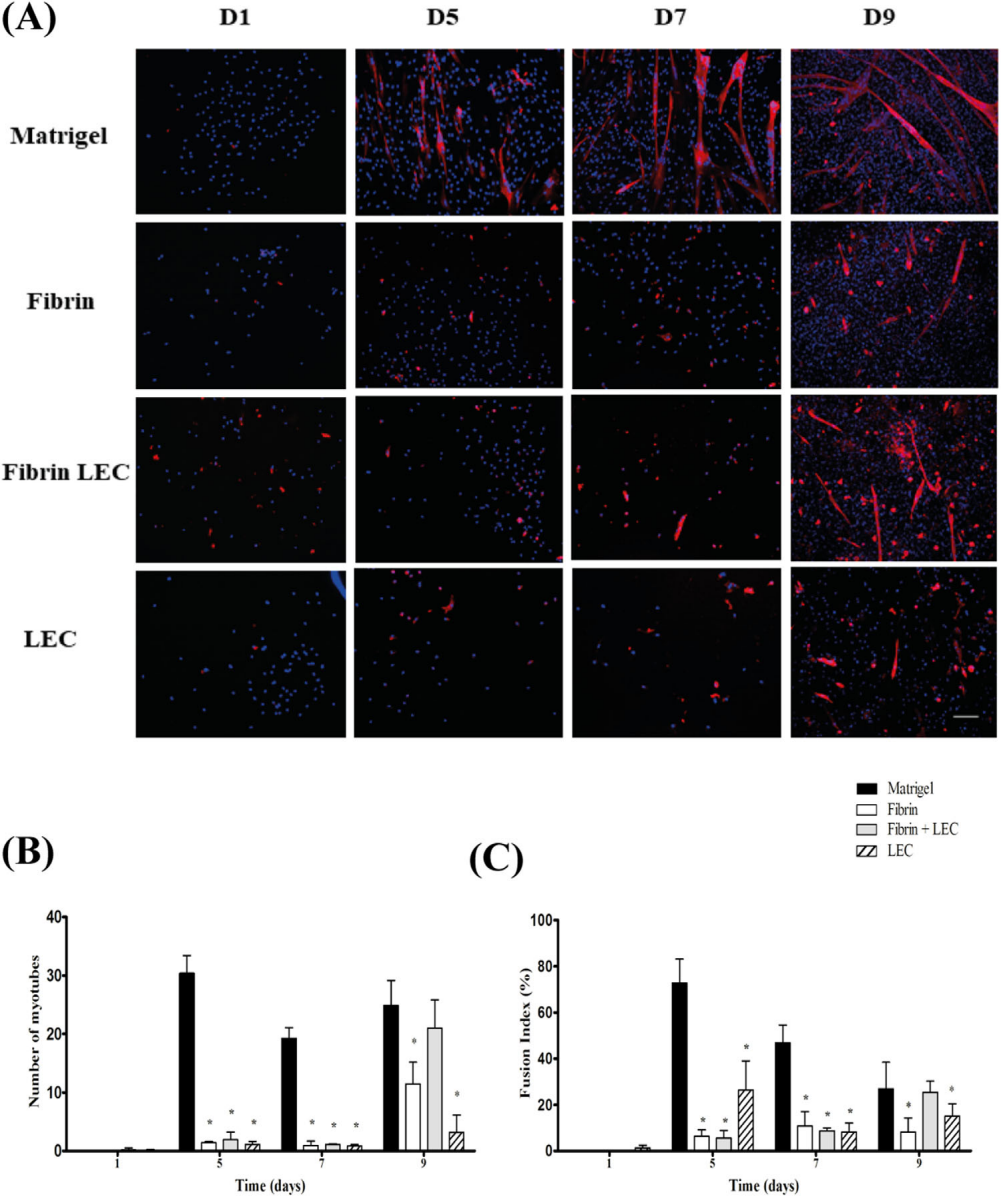
Myotube formation. (A) Representative MyHC (red) staining. SCs were seeded on matrigel, fibrin, fibrin plus LEC, and LEC alone. Scalebar represents 100 μm. (B) Number of myotubes formed and (C) fusion index per coating overtime. LEC, laminin‐entactin complex. *indicates a statistically significant difference for the coating as compared to Matrigel, according to the multiple regression analysis

Satellite cells were seeded on matrigel, fibrin, fibrin plus LEC and LEC alone and stained for Myosin Heavy Chain (MyHC) to visualize myotubes (Figure 4A). On matrigel, myotubes start forming at day 5. On the other coatings no myotubes were present at day 5. On fibrin alone and LEC alone, a low number of myotubes are formed at day 9. On fibrin plus LEC, the number of myotubes seems to be lower than on matrigel at day 9.

The number of myotubes was quantified in all cultures. A myotube is defined as a MyHC‐positive cell with at least two nuclei. On matrigel, no myotubes were present at day 1, but the average number increased to 30.4 [SD 3.0] at day 5. Thereafter, it remained at this level. Also, for fibrin, fibrin plus LEC and LEC alone, there were no myotubes at day 1. The statistical analysis showed interactions with time (Table S3). For all coatings, the number of myotubes was lower than seen on matrigel at days 5 and 7 (all *p* < 0.05). At day 9 the number of myotubes was increased on fibrin (11.5 [SD 3.7]), on fibrin plus LEC (21.0 [SD 4.8]), and on LEC alone (3.2 [SD 2.9]), though all counts remained lower than matrigel (24.9 [SD 4.3]) (*p* < 0.05). The number of myotubes on fibrin plus LEC was higher than on the other coatings (*p* < 0.05).

The fusion index was calculated as the percentage of nuclei located inside the myotubes (Figure 4C). At day 1, the fusion index was around zero for all coatings. The regression analysis showed interactions with time (Table S3). The fusion index on matrigel increased to 72.7 [SD 10.3] at day 5 and then decreased until day 9. The total number of DAPI positive cells in matrigel increased extensively over time, and these are partly fibroblasts as shown by immune staining in separate experiments. As fibroblasts do not contribute to the fusion, the fusion index at days 7 and 9 was lower. On both fibrin and LEC alone, the index was lower at days 5, 7, and 9 (all *p* < 0.05). On fibrin plus LEC, the fusion index was similar to that on matrigel at day 9 (25.5 [SD 4.8] vs 26.9 [SD 11.5]).

## DISCUSSION

The aim of this study was to analyze the effects of fibrin coatings enhanced with basement membrane components on the differentiation of satellite cell from head muscles. Fibrin‐based materials may be suitable to improve the regeneration of muscles in the soft palate after reconstructive surgery in cleft palate patients. This condition limits the function of the soft palate, especially in speech development with serious impact on social interaction and well‐being [12, 15, 51, 52]. In the field of regenerative medicine, in vitro and in vivo research is conducted to improve muscle regeneration using satellite cells, scaffolds, and growth factors [13]. In the oral cavity, fibrin hydrogels would be a suitable material as it does not require bandages to remain in place. In the present study we evaluated the attachment and differentiation of satellite cells (SCs) from the masseter muscle on fibrin coatings with and without basal lamina components in vitro. We used matrigel coatings as a positive control, as this is a standard substrate for myoblast cultures but cannot be applied clinically. Fibrin was chosen for its biocompatibility and previous applications in vivo and in vitro in regenerative medicine [12, 15, 19, 20, 21, 23, 24, 25, 26, 27, 28, 29, 32, 53]. SCs were isolated from the masseter muscle of rats, which is largely comparable to the human masseter [54, 55, 56]. Laminin, collagen IV, and laminin‐entactin complex were used to enhance fibrin as these are the main components of matrigel and also part of the SC niche. The concentrations are based on a previous study on SCs from the extensor digitorum longus muscle in mice [39, 57].

Our initial results demonstrated that Pax7‐positive SCs attach less to fibrin after one day of culture, while matrigel showed a high cell attachment. Fibrin contains binding sites for the integrin αIIbβ3, which allows platelet aggregation [58, 59]. Platelets bind to the RGD peptides identified in fibrinogen [60]. After clotting, the fibrin associates with other extracellular matrix (ECM) components such as collagen [61]. Then, fibroblasts can adhere to and migrate through the matrix with integrins such as αvβ3, α5β1, and αvβ5. This also allows the migration of endothelial cells and smooth muscle cells [58, 59, 61]. The integrin expression pattern of SCs and myoblasts changes during myogenic differentiation [62]. During the development of trunk and limb muscles, the SCs express the integrins α1β1, α2β1, α4β1, and α5β1 that bind collagen, laminin, and fibronectin. Differentiating myoblasts express the integrins α1β1 and αvβ1 to bind to fibronectin and vitronectin in the provisional ECM [62]. The myofibers in trunk and limb muscles express the integrins α6β1 and α7β1 that bind to the laminins in the basement membrane of muscle tissue [62]. Human craniofacial myoblasts additionally express the integrins α3β1, α5β1, and αvβ1 during differentiation in order to bind to laminin 111, laminin 211, vitronectin, and fibronectin [63, 64]. Myofibers from the head express the integrins α7β1, α11β1, α5β1, αvβ5, and αvβ3 that bind to the laminins in the basement membrane and also to fibronectin, vitronectin, tenascin, and collagen [48, 63]. Entactin binds to the integrins α3β1 and αvβ3, and is able to interact with fibrin through the alpha and beta chains in fibrinogen [65, 66, 67]. Similar to fibroblasts, SCs bind to fibrin through attachment to the associated ECM components. This explains that fibrin requires additional ECM components to enhance SC adhesion and differentiation.

The addition of collagen IV did not improve the attachment of SCs to fibrin. Of the analyzed coatings, collagen IV contains binding sites for integrin α1β1 only expressed at low levels by SCs from the head [63, 64]. Also, it contains binding sites for integrin α11β1, which allows the binding of mature myofibers [64]. On the other hand, the laminin coating and the coating formed by laminin and entactin interact with SCs via the integrins mentioned above [48, 63, 64]. Laminin 521 binds to the integrin α7β1 that in the SC niche facilitates self‐renewal and maintains their cell differentiation ability [48]. Yet, in our experiments the number of cells attached to LN521 was lower than in matrigel. We also observed in our experiments that the addition of laminin‐entactin to fibrin increased SC attachment. The coating with laminin‐entactin alone contains the laminins LN111 and LN211, which also occur in matrigel. The synergic effect of the laminins and the entactin enhances cell attachment. Matrigel is a standard substrate for in vitro studies of what is useful as a reference coating. The finding that myoblasts do not attach well to pure fibrin is highly relevant for possible later clinical application. Fibrin alone is not so suitable for allowing myoblasts to enter an implanted construct, which may be improved by adding basal lamina components.

Our subsequent experiments focused on the proliferation, differentiation and myotube formation of SCs on selected coatings based on the attachment data. We determined the proliferation index of SCs cultured on matrigel, fibrin, fibrin plus laminin‐entactin complex, and laminin‐entactin alone in order to estimate the proliferative activity of the cells [51]. On matrigel, the proliferation index seemed to increase gradually. However, on fibrin alone, the proliferation index was much lower than on matrigel. This might be due to the lack of binding sites for SCs in fibrin. Interestingly, the proliferation index for laminin‐entactin complex with and without fibrin was mostly similar to that of matrigel. The increase of the proliferation index on matrigel was also shown previously for head SCs from both mice and rats [32, 43, 51, 53]. This proliferative stage seems to be regulated by niche factors that become available after injury that are also present in matrigel, such as laminin, collagen IV, and entactin. Subsequently, to estimate the differentiation of SCs on our coatings, we determined the differentiation index [51]. The differentiation index of SCs cultured on fibrin alone was much lower than on matrigel. This might also be explained by the initial poor attachment of SCs to fibrin. A previous study also showed a low attachment from murine tibialis muscle derived‐SCs on fibrin coatings, as well as only limited differentiation when implanted in a muscle injury [33]. On the contrary, the differentiation index on both fibrin plus laminin‐entactin and laminin‐entactin alone was similar to that of matrigel. Another study using laminin‐entactin plus crosslinked collagen type‐I as a coating also showed enhanced proliferation and differentiation of murine derived primary myoblasts of the extensor digitorum longus muscle [39].

Finally, the number of myotubes formed on matrigel increased strongly, while on fibrin alone the number of myotubes remained low. Other studies also show that contractile myotubes formed on matrigel from cultured murine limb‐cells and head muscle‐derived SCs [43, 51]. For fibrin plus laminin‐entactin complex and laminin‐entactin complex alone, the myotubes started to form later but eventually the number of formed myotubes was similar. In short, we were able to stimulate myotube formation on fibrin coatings by adding the basal lamina components laminin and entactin. Our results may contribute to the development of strategies to reduce fibrosis, improve muscle regeneration, and improve the treatment of clinical conditions that imply extensive muscle loss. These conditions include reconstructive surgery after muscle trauma and the repaired soft palate of cleft palate patients.

In summary, our experiments were performed to contribute to the improvement of soft palate regeneration in patients after surgery for cleft lip and palate and cleft soft palate. Fibrin is a biocompatible material that is already used clinically during surgery as tissue glue. Enhanced fibrin hydrogels with key SCs niche components such as laminin and entactin are promising biomaterials for muscle regeneration. SCs from branchiomeric muscles differ from those in trunk and limb muscles. SCs from the head proliferate more and differentiate at a slower rate than other muscles. In this study, the addition of laminin‐entactin complex improved the attachment and differentiation of rat head SCs on a fibrin coating. Further research in animal models is required to evaluate the efficacy in vivo.

## CONFLICT OF INTEREST

The authors confirm that there are no known conflicts of interest associated with this publication.

## AUTHOR CONTRIBUTIONS

**Conceptualization**: Johannes W. Von den Hoff; **Methodology**: Olivier Lijten, Doris Rosero Salazar; **Formal analysis**: Olivier Lijten, Doris Rosero Salazar, Merijn van Erp, Ewald Bronkhorst; **Original draft preparation**: Olivier Lijten, Doris Rosero Salazar; **Review and editing**: Doris Rosero Salazar, Ewald Bronkhorst and Johannes W. Von den Hoff: **Project administration**: Johannes W. Von den Hoff. All authors approved the final version of the manuscript.

## Supporting information

Supporting Information
