# Supplementary material for: Effect of niche components on masseter satellite cell differentiation on fibrin coatings
Source: Eur J Oral Sci. 2022 Jan 12;130(2):e12849. doi: 10.1111/eos.12849 (PMC9303748; doi:10.1111/eos.12849)
Supplement: Supplementary file 1 — Supporting Information [file EOS-130-0-s001.pdf]

SUPPORTING INFORMATION

Effect of niche components on masseter satellite cell differentiation on fibrin coatings

Olivier Willem Lijten, Doris Haydee Rosero Salazar, Merijn van Erp, Ewald Bronkhorst, Johannes W. Von den Hoff

Radboud University Medical Center, Nijmegen, The Netherlands

Faculty of Health, Universidad Icesi, Cali, Colombia

Tables S1, S2, and S3, complement the statistical calculations of the multivariable linear regression analysis for each staining.

Table S1. Pax7/MyoD double staining

(A)

**Pax7%**	Effect	P-value	95% ci
(Intercept)	72.806	<0.001	[52,486...93,125]
Time	-3.525	0.034	[-6,779...-0,271]
Fibrin vs Matrigel	-65.790	<0.001	[-94,525...-37,054]
Fibrin + LEC vs Matrigel	3.972	0.781	[-24,764...32,707]
LEC vs Matrigel	-3.438	0.81	[-32,173...25,298]
Time*Fibrin	6.380	0.008	[1,778...10,981]
Time*Fibrin + LEC	-1.410	0.539	[-6,011...3,191]
Time*LEC	-0.133	0.954	[-4,734...4,469]

(B)

**MyoD%**	Effect	P-value	95% ci
(Intercept)	36.190	<0.001	[23,787...48,593]
Time	0.549	0.475	[-0,986...2,084]
Fibrin vs Matrigel	-24.079	<0.001	[-36,925...-11,233]
Fibrin + LEC vs Matrigel	-3.238	0.614	[-16,085...9,608]
LEC vs Matrigel	-7.016	0.277	[-19,863...5,83]

(C)

**ProlIferation index**	Effect	P-value	95% ci
(Intercept)	35.856	<0.001	[21,498...50,213]
Time	1.648	0.068	[-0,129...3,425]
Fibrin vs Matrigel	-27.687	0.001	[-42,557...-12,816]
Fibrin + LEC vs Matrigel	-11.565	0.124	[-26,436...3,305]
LEC vs Matrigel	-3.716	0.617	[-18,587...11,155]

Table S2. MyoD/MyoG double staining

(A)

**MyoD%**	Effect	P-value	95% ci
(Intercept)	35.785	<0.001	[24,281...47,289]
Time	1.047	0.146	[-0,377...2,471]
Fibrin vs Matrigel	-21.253	0.001	[-33,169...-9,337]
Fibrin + LEC vs Matrigel	-5.611	0.348	[-17,527...6,305]
LEC vs Matrigel	-9.578	0.112	[-21,494...2,338]

(B)

**MyoG%**	Effect	P-value	95% ci
(Intercept)	24.751	0.008	[6,972...42,53]
Time	0.916	0.519	[-1,931...3,763]
Fibrin vs Matrigel	-16.112	0.203	[-41,255...9,031]
Fibrin + LEC vs Matrigel	23.933	0.062	[-1,21...49,077]
LEC vs Matrigel	25.391	0.048	[0,248...50,535]
Time*Fibrin	0.458	0.819	[-3,568...4,484]
Time*Fibrin + LEC	-2.599	0.199	[-6,625...1,427]
Time*LEC	-3.506	0.086	[-7,532...0,52]

(C)

**Differentiation index**	Effect	P-value	95% ci
(Intercept)	26.782	<0.001	[19,569...33,994]
Time	0.287	0.52	[-0,606...1,18]
Fibrin vs Matrigel	-13.819	0.001	[-21,29...-6,349]
Fibrin + LEC vs Matrigel	2.479	0.507	[-4,991...9,949]
LEC vs Matrigel	0.914	0.806	[-6,556...8,384]

Table S3. MyHC staining

(A)

**Number of myotubes**	Effect	P-value	95% ci
(Intercept)	34.528	<0.001	[20,891...48,165]
Time	-1.383	0.146	[-3,281...0,514]
Fibrin vs Matrigel	-47.544	<0.001	[-66,83...-28,259]
Fibrin + LEC vs Matrigel	-59.872	<0.001	[-79,158...-40,587]
LEC vs Matrigel	-36.411	0.001	[-55,697...-17,126]
Time*Fibrin	3.900	0.006	[1,217...6,583]
Time*Fibrin + LEC	6.150	<0.001	[3,467...8,833]
Time*LEC	1.900	0.158	[-0,783...4,583]

(B)

**Fusion index**	Effect	P-value	95% ci
(Intercept)	129.035	<0.001	[105,699...152,371]
Time	-11.458	<0.001	[-14,705...-8,212]
Fibrin vs Matrigel	-123.597	<0.001	[-156,6...-90,595]
Fibrin + LEC vs Matrigel	-150.550	<0.001	[-183,553...-117,548]
LEC vs Matrigel	-92.736	<0.001	[-125,739...-59,734]
Time*Fibrin	11.892	<0.001	[7,3...16,483]
Time*Fibrin + LEC	16.428	<0.001	[11,836...21,019]
Time*LEC	8.642	0.001	[4,051...13,234]
